# Lymphatic embolization for the management of symptomatic pelvic lymphocele after radical prostatectomy with lymph node dissection: Report of two cases

**DOI:** 10.1002/iju5.12212

**Published:** 2020-10-07

**Authors:** Gregoire Schneider, Said Ourfali, Olivier Rouviere, Gaele Pagnoux, Marc Colombel

**Affiliations:** ^1^ Urologic Surgery and Transplantation Department Hôpital Edouard Herriot Hospices Civils de Lyon Lyon France; ^2^ Department of Uroradiology Hôpital Edouard Herriot Hospices Civils de Lyon Lyon France; ^3^ Université Lyon 1 Faculté de Médecine Lyon Est Université de Lyon Lyon France

**Keywords:** lymph node dissection, lymphatic embolization, pelvic lymphocele, radical prostatectomy

## Abstract

**Introduction:**

Pelvic lymphocele is the most common complication of pelvic lymph node dissection after radical prostatectomy. Management of symptomatic pelvic lymphocele begins with percutaneous drainage, followed by sclerotherapy or surgical marsupialization and more recently, lymphatic embolization. In this article, we show the feasibility and results of two lymphatic embolization after prostatectomy with lymph node dissection.

**Case presentation:**

We decided to perform lymphatic embolization in two patients with persistent symptomatic pelvic lymphocele, after percutaneous drainage. This was done through inguinal lymph node puncture using Lipiodol and *N*‐butyl cyanoacrylate glue injection. Drainage removal was done on the day after the procedure and clinical recovery was maintained at follow‐up visits, 3 and 4 months later, in both patients. Computed tomography at 6 and 10 weeks after embolization showed the disappearance of the lymphocele.

**Conclusion:**

Our two case reports support the promising results of lymphatic embolization in this pathology.

**Abbreviations & Acronyms**
CT: computed tomography
PL: pelvic lymphocele
PLND: pelvic lymph node dissection
RP: radical prostatectomy

**Keynote message**

Symptomatic PL is a common complication after RP with PLND. We present here the feasibility and positive outcome of lymphatic embolization in two patients. This technique is a promising therapy in the management of this complication.

## Introduction

RP with PLND is a therapeutic option in high‐risk prostate cancer.^1^ Although PLND does not improve survival or oncologic outcomes, it improves the staging of the disease, which may influence the postoperative treatment strategy.^2^ However, the risk of postoperative complications is significantly increased.^2^, ^3^

PL is the most common complication of PLND after RP. Up to 11% become symptomatic and lead to complications such as infection, pelvic pain, and deep vein thrombosis.^2^, ^4^, ^5^ Management of these symptomatic PL usually begins with percutaneous drainage, followed by sclerotherapy or/and surgical marsupialization.^6^

Lymphangiography and lymphatic embolization with *N*‐butyl cyanoacrylate glue are emerging strategies that have been used to treat postoperative lymphoceles after hysterectomy, kidney transplantation and, more recently, prostatectomy.^7^, ^8^, ^9^

We report two cases of lymphatic embolization after RP with PLND.

## Case presentation

We report the case of two patients presenting with a symptomatic lymphocele in the immediate follow‐up of extraperitoneal robotic prostatectomy and PLND using clips and bipolar forceps. These lymphoceles were initially drained percutaneously. Given the persistence of a productive drain (>200 mL/day), lymphangiography and lymphatic embolization were done.

First, a direct inguinal lymph node puncture, ipsilateral to the lymphocele, was performed under ultrasound guidance with a 22‐gauge needle. Lymphangiography was obtained by injecting manually Lipiodol™ Ultra Fluid (Guerbet, Aulnay‐sous‐Bois, France) in the lymph node (0.5 mL/min maximum flow). Opacification of the lymphatic ducts and nodes was observed under fluoroscopy until the site of lymphatic leakage was identified as Lipiodol extravasation. Then, the lymph duct or node involved in the leak was punctured under fluoroscopic guidance and embolized by injecting 1 cc of 1:9 mixture of *N*‐butyl cyanoacrylate‐methacryloxy sulfolane (Glubran™ 2; GEM Srl, Viareggio, Italy) glue and Lipiodol™ Ultra Fluid.

Patients were hospitalized until lymphatic leakage was inferior to 50 cc/day, and the drain was removed. Patients were followed at 1 and 4 months. Informed consent was obtained from both patients.

### Patient 1

**Fig. 1:**
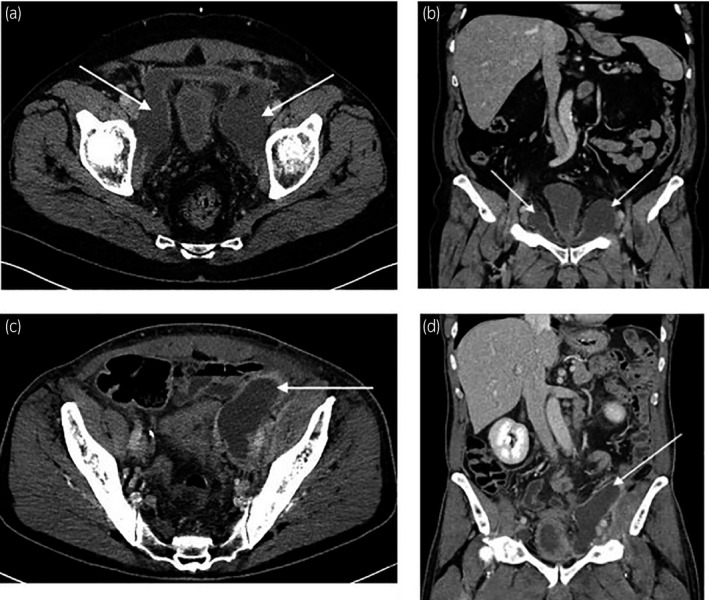
Before lymphatic embolization. (a, b) Patient 1: CT scanner at day 10 after RP, before percutaneous drainage (a, axial slice; b, coronal reformation). PL indicated by the arrow. (c, d) Patient 2: CT scanner showing a voluminous left PL (c, axial slice; d, coronal reformation). Compression of the external iliac vein. PL indicated by the arrow.

A 67‐year‐old patient presenting a symptomatic PL, at day 10 postsurgery. The patient’s complaint was abdominal pain with fever. CT scanner showed a large anterior bilateral pelvic collection (Fig. 1a,b).

Antibiotherapy and percutaneous drainage were first carried out but the daily flow continued to exceed 500 mL/day, even after 1 week.

**Fig. 2:**
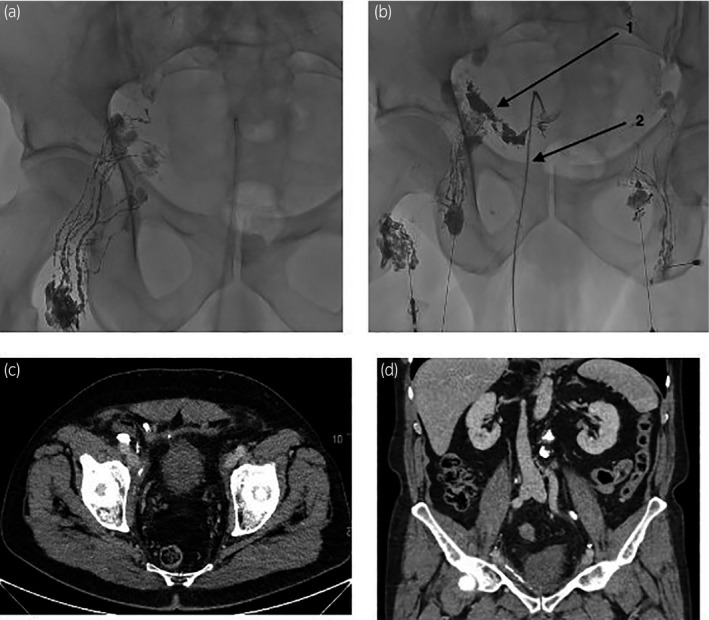
Patient 1: lymphangiography and follow‐up. (a) Right lymphangiography: no leak initially identified. (b) During left lymphangiography: leakage (1) on the right side showed on late images. The leak is directed toward the percutaneous catheter (2), and probably comes from external iliac lymph ducts. Puncture and glue/Lipiodol injection into the closest lymph node from the leak. (c, d) CT scan 10 weeks after the right lymphatic embolization (a, axial slice; b, coronal reformation). No residual lymphocele.

Lymphangiography started on the right side. No leak was identified on early images. Left side lymphangiography was done without lymphatic leakage, but late images revealed a leak on the right side (Fig. 2).

Since no lymphatic canal was directly accessible, lymphatic embolization was done through the lymph node closest to the leak.

The drainage amount decreased after embolization (20 mL/24 hours), allowing the removal of the drainage catheter on day 1. The patient returned home on day 2. At follow‐up visits at 1 and 4 months, the patient remained asymptomatic. The CT scanner performed 10 weeks after lymphatic embolization showed a complete regression of the lymphocele (Fig. 2).

### Patient 2

A 72‐year‐old patient presenting fever, abdominal pain, and left leg lymphoedema on day 29 postoperatively.

The CT scanner showed a voluminous and compressive lymphocele on the left side of the pelvis (Fig. 1c,d). After 10 days of productive drainage (>500 mL/day), the patient was referred for lymphatic embolization.

**Fig. 3:**
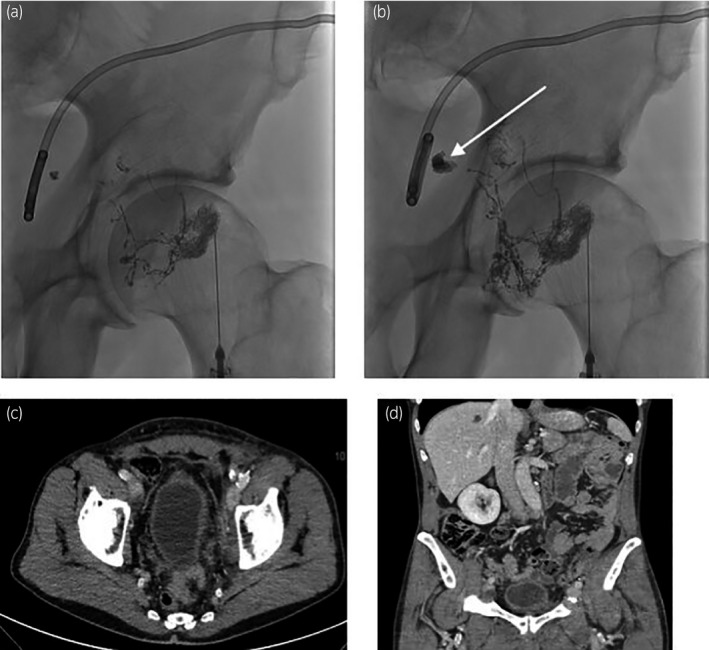
Patient 2: lymphangiography and follow‐up. (a) Left inguinal lymphangiography. (b) Later images showing a leakage from external iliac lymph ducts next to the percutaneous catheter, followed by a glue injection into the closest lymph node. (c, d) CT scan 6 weeks after the left lymphatic embolization. No residual lymphocele.

The lymphatic leakage was located next to the drainage catheter (Fig. 3), and embolization was performed through the closest lymph node.

No liquid was collected during the next 24 hours and the drainage catheter was removed the day after the procedure. The patient was discharged after catheter removal.

At follow‐up visits at 1 and 3 months, the lymphoedema had regressed significantly, with lymphatic drainage physiotherapy. A CT scanner was performed 6 weeks after embolization and showed no residual collection (Fig. 3).

### Results

One single embolization was sufficient. Hospitalization time was 2 days for both patients. There was neither recurrence nor postprocedure complications during the follow‐up (Table 1).

**Table 1:** Patients’ characteristics and results

Pt	Age	Symptoms	Drainage (mL/day)	Lymphatic embolization (number of embolization)	Glue amount needed (cc)	Time of procedure (min)	Drainage post‐embolization (mL/day)	Time to drain removal (day)	Clinical success	Radiological success	Follow‐up (months)
1	67	Abdominal pain and fever	>500	Intranodal (1)	1	–	20	1	Yes	Yes	4
2	72	Painful bilateral leg edema and sepsis	>500	Intranodal (1)	1	–	0	1	Yes	Yes	3

Drainage: amount of lymph produced by the drain before embolization. Drainage post‐embolization: amount of lymph produced by the drain after embolization. Clinical success is defined by the resolution of symptoms related to the lymphocele and a catheter drain producing less than 10cc/day, allowing its removal. Radiological success is defined by the disappearance of the collection at the CT scan at least 6 weeks later.

## Discussion

In these two cases, lymphatic embolization was effective to treat symptomatic lymphoceles, after RP with PLND, for which percutaneous drainage was insufficient. This minimally invasive procedure resulted in rapid recovery, with removal of the drainage catheter and patient discharge the following day. Although a longer follow‐up is necessary to ensure that there will be no recurrence, our results are interesting.

Percutaneous catheter drainage is a conservative way to treat symptomatic lymphoceles. However, retrospective studies report the persistence of lymphatic leakage in 23–50% with recurrence.^10^ At this stage, a known option is sclerotherapy, which consists of an injection of sclerosing products such as alcohol, povidone‐iodine, and fibrin sealants through the drainage catheter. Success rates vary from 70 to 100%. There is a 20–25% risk of recurrence, which can be resolved by another sclerotherapy session. However, the drains are kept for a mean range of 10–20 days.^6^, ^11^, ^12^ Another more radical option is marsupialization. This surgical procedure offers low morbidity, fast recovery, and a low recurrence rate (<15%).^13^, ^14^

Lymphatic embolization has been an emerging technique for several years to treat PL. Baek *et al*. reported a 95% efficacy in a series of 21 patients after gynecological surgery. Efficacy was obtained by one or more embolizations with a mean duration of hospitalization of 5.9 days.^15^ Chu *et al*. showed 100% of clinical and technical efficacy in nine patients with symptomatic PL after RP and PLND. Six patients required a single embolization with an average drain removal time of 7 days. The remaining three patients required a second embolization due to additional leakage not visible on the first lymphangiography.^9^

The technical aspects of the procedure were similar to these two series, showing good reproducibility.^9^, ^15^ Puncture was done with a larger needle in our cases (22‐gauge compared to 25). This can be adapted to the size of nodes. The same substances were used. We injected a more diluted mixture of glue with Lipiodol (1:9 *vs* 1:2 to 1:9), in order to avoid early polymerization of the glue to reach the leak.

No adverse events following this type of procedure, neither major nor minor, have been reported.^9^, ^15^

This technique, less invasive than surgical marsupialization, appears to give a good and similar efficiency. Compared to sclerotherapy, it seems to allow a quicker catheter drainage removal and faster recovery.^6^, ^9^, ^11^, ^15^ Our data support previous results of lymphatic embolization for treatment of PL after RP and PLND.

## Conclusion

Lymphatic embolization is an emerging and promising therapy in this pathology. Our data concern only two patients with a short follow‐up and studies on a large number of patients are expected.

## Conflict of interest

The authors declare no conflict of interest.
